# Resveratrol Attenuates Oxidative Stress-Induced Intestinal Barrier Injury through PI3K/Akt-Mediated Nrf2 Signaling Pathway

**DOI:** 10.1155/2019/7591840

**Published:** 2019-12-02

**Authors:** Yu Zhuang, Huirong Wu, Xiangxiang Wang, Jieyu He, Shanping He, Yulong Yin

**Affiliations:** ^1^Hunan International Joint Laboratory of Animal Intestinal Ecology and Health, Laboratory of Animal Nutrition and Human Health, School of Life Sciences, Hunan Normal University, Changsha, 410081 Hunan, China; ^2^Jiangxi Provincial Key Laboratory for Animal Health, Institute of Animal Population Health, College of Animal Science and Technology, Jiangxi Agricultural University, No. 1101 Zhimin Avenue, Economic and Technological Development District, Nanchang, 330045 Jiangxi, China; ^3^Human Engineering & Research Center of Animal and Poultry Science, Key Lab Agroecology Processing Subtropical Region, Scientific Observational and Experimental Station of Animal Nutrition and Feed Science in South-Central, Ministry of Agriculture, Institute of Subtropical Agriculture, Chinese Academy of Science, Changsha, 410125 Hunan, China

## Abstract

Oxidative stress is implicated in a wide range of intestinal disorders and closely associated with their pathological processes. Resveratrol (RSV), a plant extract, plays a vital role in protecting various organs *in vitro* and *in vivo*. However, the benefits of RSV are controversial, and underlying mechanisms for its antioxidant effects on intestinal epithelial cells remain unclear. In this study, we evaluated the effects of RSV on oxidative stress induced by H_2_O_2_ in IPEC-J2 cells. We found that pretreatment with RSV significantly increased cell viability; increased expression levels of tight junction (TJ) proteins (claudin-1, occludin, and ZO-1); improved activities of superoxide dismutase-1 (SOD-1), catalase (CAT), and glutathione peroxidase (GSH-Px); and decreased intracellular reactive oxygen species (ROS) levels and apoptosis induced by H_2_O_2_ (*P* < 0.05). In addition, RSV upregulated Akt phosphorylation, Nrf2 phosphorylation, and expression levels of antioxidant genes HO-1, SOD-1, and CAT in a dose-dependent manner (*P* < 0.05) under oxidative stress. Knockdown of Nrf2 by short-hairpin RNA (shRNA) abrogated RSV-mediated protection against H_2_O_2_-induced apoptosis, RSV-induced increase of TJ protein levels, and antioxidant gene expression (SOD-1, CAT, and GSH-Px) (*P* < 0.05). Consistent with Nrf2 knockdown, the PI3K/Akt inhibitor LY294002 significantly suppressed RSV-induced Nrf2 phosphorylation and RSV-induced increase of TJ protein levels and antioxidant gene expression under H_2_O_2_ treatment (*P* < 0.05). Collectively, these results demonstrate that RSV can directly protect IPEC-J2 cells against oxidative stress through the PI3K/Akt-mediated Nrf2 signaling pathway, suggesting that RSV may be an effective feed additive against intestinal damage in livestock production.

## 1. Introduction

The intestine not only is a major digestive and absorptive organ for nutrients but also functions as a selective barrier against toxins, pathogens, and antigens from the luminal environment [1]. The intestinal barrier primarily consists of tight junction proteins (TJs), enterocyte membrane, antibacterial peptides, and the mucous layer and immune system. When the intestinal barrier is disrupted, the luminal toxins and antigens will penetrate subepithelial tissues through the barrier, causing a mucosal oxidative stress and systemic inflammatory response [1]. Meanwhile, overproduction of reactive oxygen species (ROS) and proinflammatory cytokines disrupts the intestinal barrier and dysfunction. However, due to the complex physiological and/or chemical environment of the intestine, it is extremely susceptible to oxidative stress.

Oxidative stress, defined as the imbalance between the antioxidant systems and oxidative system causing overdose of ROS, can disrupt cellular signaling and function. It is believed that oxidative stress is involved in the development of intestinal diseases such as inflammatory bowel disease (IBD), irritable bowel syndrome, and colon cancer [2–5]. Under the physiological condition, ROS is maintained at certain levels and excessive free radicals are usually scavenged by antioxidant enzymes such as superoxide dismutase (SOD), catalase (CAT), and glutathione peroxidase (GPH-Px). However, under the imbalance between the antioxidant and the oxidative system, overdose of ROS can disturb epithelial cell integrity and intestinal barrier by decreasing tight junctions and cell quantity [6]. Recent studies have shown that ROS or other free radicals can disturb cell functions by influencing transcription factors and the redox-sensitive signaling pathway. Nuclear factor erythroid 2-related factor 2 (Nrf2) is a transcription factor that has an important regulative effect on oxidative statues through induction of the expression of the antioxidant and phase 2 detoxifying enzymes and related proteins [7, 8]. In terms of the possible importance of ROS in intestinal injury, it is essential for cells to effectively upregulate antioxidants, decrease ROS production, and scavenge free radicals, which may contribute to increased intestinal permeability and epithelial apoptosis.

Plant extracts are considered as a potential source of antioxidant and anti-inflammatory molecules which have been identified and proposed as therapeutic agents to counteract oxidative stress-related disease [9]. Resveratrol (RSV) is a plant-derived stilbene (polyphenol) associated with a wide range of health benefits [10–13]. Many studies have suggested that RSV acts on multiple cellular targets such as Nrf2, NF-K*β*, Sirt1, and AMPK to control processes and signaling pathways related to reduction of oxidative stress, apoptosis, and anticancer effects [1, 14–16]. However, scientific research on healthy effects of dietary RSV has been controversial because of low bioavailability of RSV *in vivo* [17]. The doses of RSV in target tissues are extremely low and hardly reach the level of pharmacological concentration in *in vitro* studies [18]. Even though the function of RSV is still controversial, we hypothesize that RSV can directly protect intestines from oxidative stress through its rapid metabolism in intestinal cells. Therefore, we used an *in vitro* oxidative stress model induced by H_2_O_2_ to investigate whether RSV can prevent intestinal impairment. Our results provide insights for the future application of RSV as feed additives against intestinal damage in livestock production.

## 2. Materials and Methods

### 2.1. Chemicals and Reagents

Dulbecco's modified Eagle's medium (DMEM), fetal bovine serum (FBS), and antibiotics (penicillin and streptomycin) required for cell culture were obtained from Gibco (Carlsbad, CA, USA). Resveratrol (RSV) and LY294002 (the PI3K/Akt inhibitor) were obtained from Selleckchem (Houston, United States). The antibodies against Nrf2, Keap1, and *β*-actin were obtained from Abcam, the antibodies against claudin-1 and occludin from Selleckchem (Houston, United States), and the antibody against ZO-1 from Proteintech (Wuhan, China). The plasmids pLKO.1, pLKO-scramble, pCMV-DR8.9, and pCMV-VSVG were kind gifts from Prof. Pinghui Feng (University of Southern California, USA).

### 2.2. Cell Culture

The porcine intestinal epithelial cells (IPEC-J2 cells) and human embryonic kidney 293 (HEK293T) cells were kindly provided by Prof. Guoyao Wu (Texas A&M University, USA). All cells were cultured in Dulbecco's modified Eagle's medium (DMEM; Gibco) containing 10% heat-inactivated fetal bovine serum (FBS; Gibco) and maintained at 37°C with 5% CO_2_.

### 2.3. Establishment of Nrf2-Knockdown IPEC-J2 Cell Line

Short-hairpin RNAs (shRNAs) were designed using the online shRNA design tool (https://portals.broadinstitute.org/gpp/public/) and cloned into the lentiviral vector pLKO.01 via Age I/EcoR I. The plasmids pLKO-shNrf2 or pLKO-scramble, pCMV-DR8.9, and pCMV-VSVG were transfected into 293T cells to produce lentiviruses. At 48 hours posttransfection, the lentiviruses were harvested and used to infect IPEC-J2 cells in the presence of polybrene (8 *μ*g/mL). At 48 hours postinfection, the infected cells were selected and maintained with puromycin (2 *μ*g/mL). The sequences of shRNAs are shown in Table 1.

### 2.4. Cell Viability Analysis

Cell viability was evaluated using the CCK-8 assay (Dojindo, Kumamoto) according to the manufacturer's instructions. Briefly, IPEC-J2 cells were cultured in 96-well plates at a density of 8 × 10^4^ cells per well. On the following day, the cells were treated with medium containing different concentrations of H_2_O_2_ for 4 hours and then assayed for cell viability. RSV was treated for 6 hours before H_2_O_2_ treatment, and LY294002 (the PI3K inhibitor) at a concentration of 25 *μ*M was pretreated for 2 hours before H_2_O_2_ and RSV treatment [19].

### 2.5. Analysis of the Activities of T-SOD, CAT, and GSH-Px

IPEC-J2 cells were pretreated with RSV at the concentration of 0 *μ*M, 25 *μ*M, and 50 *μ*M for 6 h and then treated with or without 400 *μ*M H_2_O_2_ for 4 h. The cells in each group were lysed to determine the activities of T-SOD, CAT, and GSH-Px using commercial assay kits (Jiancheng, Nanjing, China) according to the instructions. The activities of T-SOD, CAT, and GSH-Px were calculated and expressed as U/mg protein.

### 2.6. Measurement of ROS Production

The relative levels of intracellular ROS were measured using a commercial ROS detection kit (Beyotime, China). Briefly, IPEC-J2 cells were cultured in 6-well plates for 16 h before treatment, pretreated with RSV (0 *μ*M, 25 *μ*M, and 50 *μ*M) for 4 hours, and then exposed with H_2_O_2_ (500 *μ*M) for 4 h to induce ROS production. Cells were washed twice with PBS and then incubated with 5 *μ*M 2′,7′-dichlorofluorescein diacetate (DCFHDA) for 30 min. Fluorescence intensity was measured using a fluorescence microplate reader (Tecan, Sunrise) at excitation/emission wavelengths of 525/610 nm.

### 2.7. Annexin V-FITC/Propidium Iodide (PI) Apoptosis Detection

Apoptosis was determined using the Annexin V-FITC cell apoptosis detection kit (KeyGEN, Nanjing, China). Briefly, IPEC-J2 cells were cultured in 6-well plates for 16 h before treatment, pretreated with Ly294002, and then treated with RSV (0 *μ*M, 25 *μ*M, and 50 *μ*M) or H_2_O_2_ according to the experimental design. Following each specific treatment, cells were collected, washed twice with ice-cold PBS, and then centrifuged at 2000 rpm for 5 min. Cells were resuspended in 500 *μ*L of 1x binding buffer and transferred to sterile flow cytometry glass tubes. 5 *μ*L of Annexin V-FITC and 5 *μ*L of PI were added, and samples were incubated at room temperature (25°C) in dark conditions for 10 min. Finally, flow cytometric analysis was performed according to the manufacturer's instructions (Beckman, Fullerton, California, USA).

### 2.8. Quantitative Real-Time PCR (qRT-PCR)

After the extraction of total RNA in treated cells, reverse transcription and qRT-PCR were performed as described previously. Primers were designed with the online primer design tool Primer-BLAST (https://www.ncbi.nlm.nih.gov/tools/primer-blast/index.cgi?LINK_LOC=BlastHome) according to the gene sequences of the pig, and the sequences of the primers are shown in Table 2. *β*-Actin was used to normalize the levels of target gene transcripts, and the relative abundance of gene transcripts was normalized to the values of the control treatment.

### 2.9. Western Blot Analysis

Cells were treated with the indicated agents according to the experimental design and lysed with RIPA buffer (Beyotime) to extract total cellular proteins. The concentration of proteins was quantified using a BCA protein assay kit (Beyotime, China) according to the instructions. Western blot analysis was performed as described previously. The indicated proteins were normalized to *β*-actin and analyzed using ImageJ (National Institutes of Health, Bethesda, MD, USA).

## 3. Statistical Analyses

Results from a representative of three independent experiments were expressed as means ± standard error of the mean (SEM). One-way analysis of variance (ANOVA) followed by the LSD test was used for multiple comparisons, and a value of *P* < 0.05 was accepted as statistically significant. The statistical analyses were performed by GraphPad Prism 7.

## 4. Results

### 4.1. Concentration-Dependent Effects of H_2_O_2_ and RSV on Cell Viability

**Figure 1:**
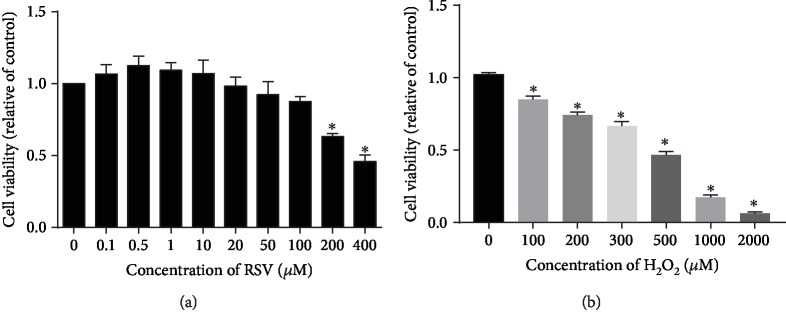
Effects of RSV and H_2_O_2_ on the viability of IPEC-J2 cells. (a) IPEC-J2 cells were incubated with increasing concentrations of RSV for 6 h (a) and H_2_O_2_ for 4 h (b), and then cell viability was measured by CCK-8 assays. Results are presented as the percentage of cell viability compared with the control (0 *μ*M). Values are the mean ± SE; *n* = 8. Asterisks indicate a significant difference compared to the control group (*P* < 0.05).

**Figure 2:**
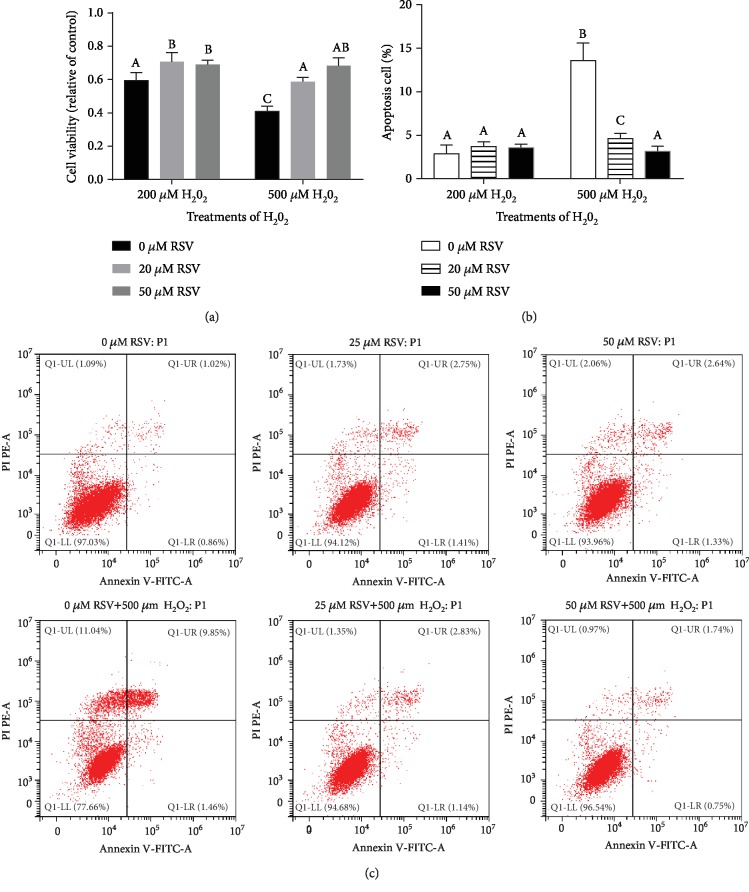
Protective effects of RSV against H_2_O_2_-induced oxidative injury in IPEC-J2 cells. IPEC-J2 cells were pretreated with the indicated concentrations of RSV and then cocultured with 200 *μ*M or 500 *μ*M H_2_O_2_ for 4 hours. (a) The CCK-8 assay was performed on IPEC-J2 cells after incubation with RSV (6 h) and H_2_O_2_ (4 h). (b, c) Apoptosis cells were analyzed by flow cytometry using Annexin V and propidium iodide (PI) double staining. Results are presented as the percentage of cell viability compared with the control (0 *μ*M). Values are the mean ± SE. The columns with the same superscript capital letters and with different superscript capital letters mean no significant difference (*P* > 0.05) and significant difference (*P* < 0.05), respectively.

To determine suitable concentrations of H_2_O_2_ and RSV for subsequent experiments, we treated IPEC-J2 cells with different concentrations of H_2_O_2_ or RSV, and measured the viability of the treated cells by CCK-8 assays. As shown in Figure 1, a high concentration of RSV showed slight inhibition on IPEC-J2 cells, and RSV significantly decreased the viability of IPEC-J2 cells at both 200 *μ*M and 400 *μ*M for 6 h (Figure 1(a)). Treatment with H_2_O_2_ for 4 h resulted in significant cell shrinkage compared to the control group. We found that a concentration of 500 *μ*M H_2_O_2_ sharply decreased cell viability to 40% compared to the control (Figure 1(b)). Pretreatment of RSV (20 *μ*M, 50 *μ*M) significantly attenuated the decreased cell viability and cell apoptosis caused by 500 *μ*M H_2_O_2_ in a dose-dependent manner (Figure 2). Based on these results, we used 500 *μ*M H_2_O_2_ to induce oxidative stress and used 20 *μ*M or 50 *μ*M RSV in subsequent experiments.

### 4.2. Resveratrol Elevates the Expression of TJ mRNAs in IPEC-J2 Cells

**Figure 3:**
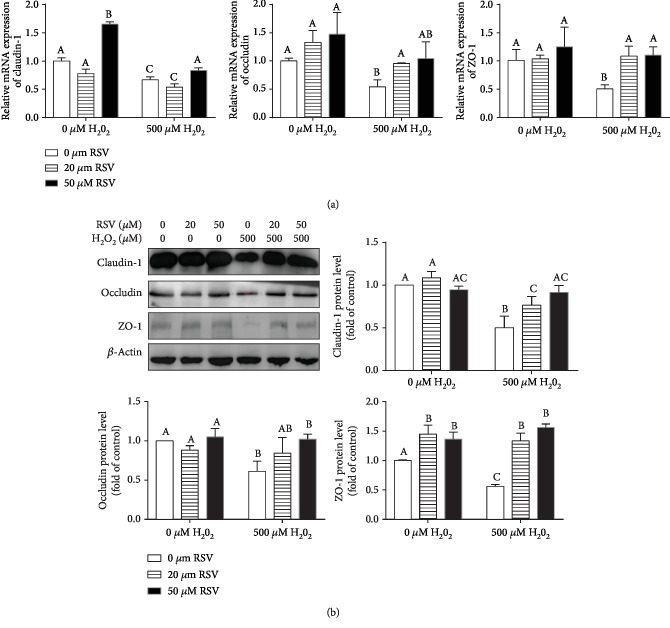
Effects of resveratrol and/or H_2_O_2_ on the levels of TJ proteins. IPEC-J2 cells were pretreated with the indicated concentrations of RSV and then cocultured with 500 *μ*M H_2_O_2_ for 4 hours. (a) The expression of claudin-1, occludin, and ZO-1 was detected by quantitative real-time PCR (qRT-PCR). Data are shown as ratios of abundance of target gene transcripts in the treated cells to those in the control cells after normalization to *β*-actin. (b) Protein levels of claudin-1, occludin, and ZO-1 were detected by Western blot with *β*-actin as the loading control. Values are the mean ± SE; *n* = 3. The columns with the same superscript capital letters and with different superscript capital letters mean no significant difference (*P* > 0.05) and significant difference (*P* < 0.05), respectively.

Intestinal integrity relies on tight junctions. Tight junctions (TJs) are the principal determinants of intestinal barrier function that seal the paracellular space between neighboring epithelial cells. Here, we examined the expression of TJ mRNAs in IPEC-J2 cell monolayers treated with or without RSV and/or H_2_O_2_. As shown in Figure 3, qRT-PCR results revealed that the expression of claudin-1, occludin, and ZO-1 was significantly decreased in IPEC-J2 cells treated with H_2_O_2_ compared with the control group (Figure 3(a)), whereas cotreatment with RSV reversed H_2_O_2_-induced downregulation of TJ proteins (claudin-1, occludin, and ZO-1). The protein levels (claudin-1, occludin, and ZO-1) examined by Western blot were consistent with the mRNA levels of TJ proteins (Figure 3(b)).

### 4.3. Resveratrol Elevates Antioxidant Ability and the Expression of Antioxidant Genes in IPEC-J2 Cells

**Figure 4:**
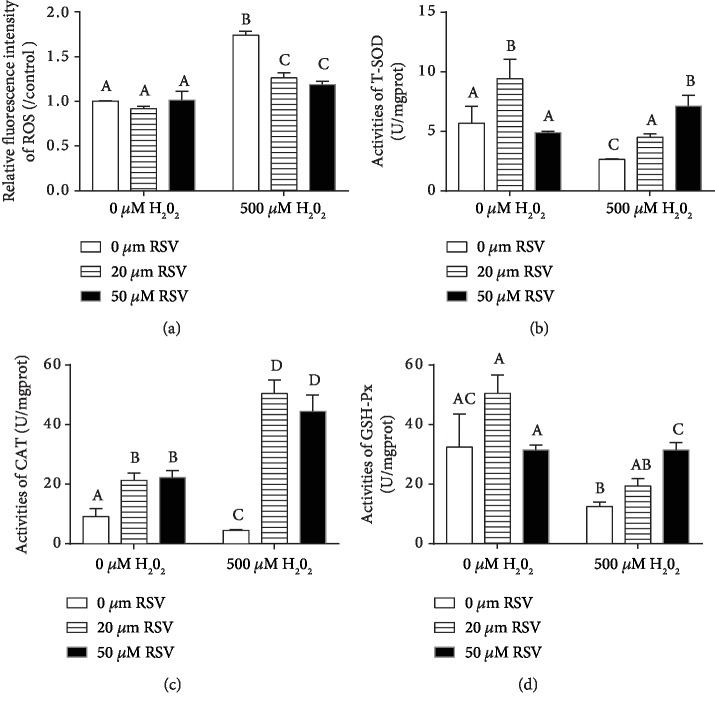
Effects of resveratrol and/or H_2_O_2_ on ROS levels and the activities of T-SOD, CAT, and GSH-Px in IPEC-J2 cells. IPEC-J2 cells were pretreated with RSV at the concentration of 0 *μ*M, 20 *μ*M, and 50 *μ*M for 6 hours and then cocultured with 500 *μ*M H_2_O_2_ for 4 hours. (a) The fluorescence intensity of ROS was measured by a fluorescence microplate reader. (b–d) Cells were pretreated with or without RSV at the indicated concentrations for 6 h and then incubated in the presence of H_2_O_2_ at the concentration of 500 *μ*M, and the activities of T-SOD (b), CAT (c), and GSH-Px (d) in cell lysates were detected by a spectrophotometer using commercial kits. Values are the mean ± SE; *n* = 6. The columns with the same superscript capital letters and with different superscript capital letters mean no significant difference (*P* > 0.05) and significant difference (*P* < 0.05), respectively.

**Figure 5:**
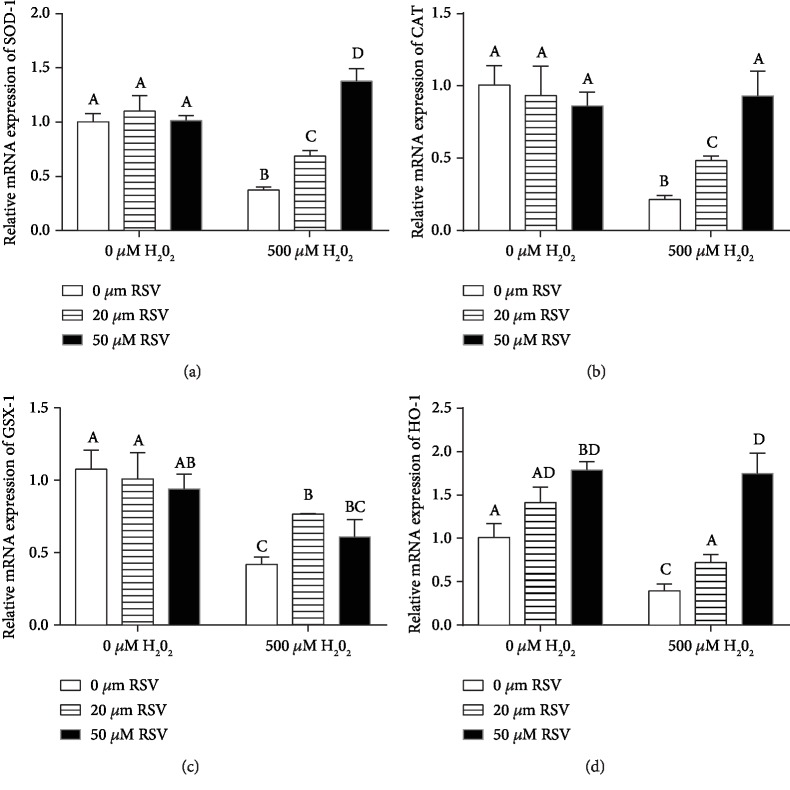
Effects of resveratrol and/or H_2_O_2_ on the expression of SOD-1, CAT, GSX-1, and HO-1 in IPEC-J2 cells. IPEC-J2 cells were pretreated with the indicated concentrations of RSV for 6 hours and then cocultured with 500 *μ*M H_2_O_2_ for 4 hours. The expression of SOD-1 (a), CAT (b), GSX-1 (c), and HO-1 (d) was detected by qRT-PCR. Data are shown as ratios of abundance of target gene transcripts in the treated cells to those in the control cells after normalization to *β*-actin. Values are the mean ± SE; *n* = 6. The columns with the same superscript capital letters and with different superscript capital letters mean no significant difference (*P* > 0.05) and significant difference (*P* < 0.05), respectively.

The activities and expression of antioxidant enzymes are the main response for relieving oxidative stress damage in organs or cells. As shown in Figure 4, no significant ROS difference was found between cells treated with RSV alone, whereas RSV significantly decreased levels of ROS in the H_2_O_2_-treated cells in a dose-dependent manner (Figure 4(a)). It was shown that RSV significantly elevated the activities of T-SOD, CAT, and GSH-Px in the cells treated with or without H_2_O_2_ (Figures 4(b)–4(d)). qRT-qPCR results revealed that high concentration of H_2_O_2_ significantly decreased the expression of SOD-1, GSX-1, catalase (CAT), and HO-1 genes, whereas RSV reversed the H_2_O_2_-induced downregulation of SOD-1, CAT, GSX-1, and HO-1 genes (Figures 5(a)–5(d)).

### 4.4. Resveratrol Protects IPEC-J2 Cells against H_2_O_2_-Induced Barrier Dysfunction via the Nrf2/Keap1 Pathway

**Figure 6:**
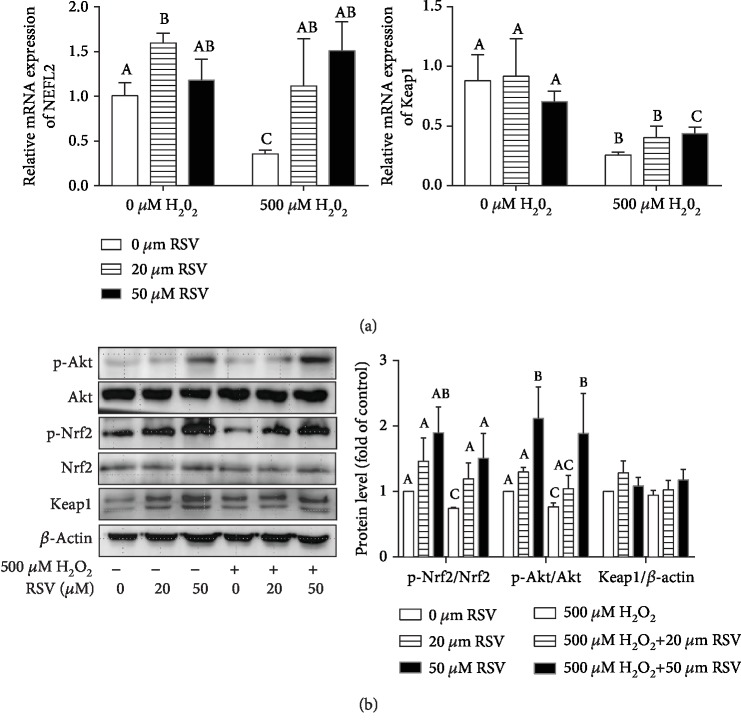
Effects of resveratrol and/or H_2_O_2_ on the Akt and Nrf2/Keap1 pathways in IPEC-J2 cells. IPEC-J2 cells were pretreated with the indicated concentrations of RSV for 6 hours and then cocultured with 500 *μ*M H_2_O_2_ for 4 hours. (a) The expression of Nrf2 and Keap1 was detected by qRT-PCR. Data are shown as ratios of abundance of target gene transcripts in the treated cells to those in the control cells after normalization to *β*-actin. (b) Protein levels of Keap1, Nrf2, Akt, p-Nrf2, and p-Akt were detected by Western blot with *β*-actin as the loading control. (b) The levels of proteins were quantified, and data are presented as the means + SD. The columns with the same superscript capital letters and with different superscript capital letters mean no significant difference (*P* > 0.05) and significant difference (*P* < 0.05), respectively.

Given that RSV is a potent antioxidative agent promoting the expression of antioxidant genes, we investigated whether RSV relieves oxidative stress through the Akt and Nrf2/Keap1 signaling pathway, which is a key cellular cascade involved in cell survival and oxidative stress response. qRT-qPCR results revealed that H_2_O_2_ treatment significantly decreased the expression of Nrf2 and Keap1 genes, whereas RSV significantly reversed H_2_O_2_-induced downregulation of Nrf2 and Keap1 genes (Figure 6(a)). Western blot analysis showed that RSV enhanced the phosphorylation of Nrf2 and Akt in a dose-dependent manner (*P* < 0.05), whereas no significant changes were found in the protein levels of Nrf2, Keap1, and Akt (Figure 6(b)).

### 4.5. Role of Nrf2 on RSV-Induced Cytoprotection against Oxidative Stress

**Figure 7:**
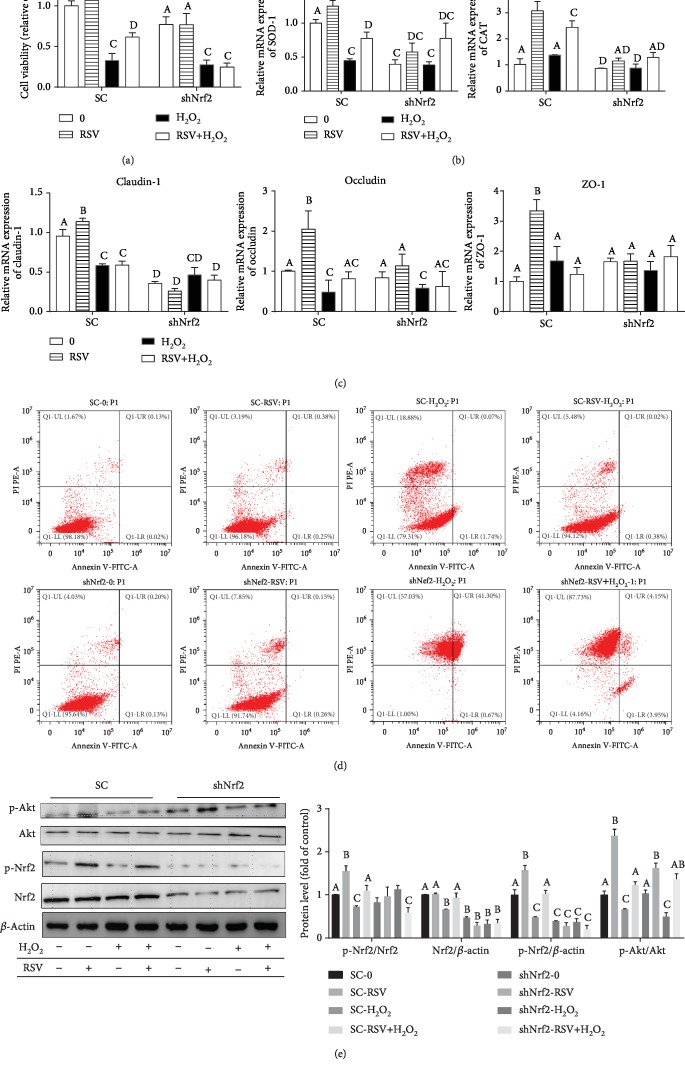
Effects of resveratrol on H_2_O_2_-induced cytotoxicity in Nrf2-knockdown IPEC-J2 cells. The Nrf2-knockdown (shNRF2-IPEC-J2) or control (SC-IPEC-J2) cells were pretreated with or without 50 *μ*M RSV for 6 hours and then cocultured with 500 *μ*M H_2_O_2_ for 4 hours. (a) Cell viability was measured using the CCK-8 assay. Results are presented as the percentage of cell viability compared with the control (0 *μ*M). (b, c) The relative expression of SOD-1, CAT, claudin-1, occludin, and ZO-1 was detected by qRT-PCR. Data are shown as ratios of abundance of target gene transcripts in the treated cells to those in the control cells after normalization to *β*-actin. (d) Apoptotic cells were analyzed by flow cytometry using Annexin V-PI double staining. (e) Protein levels of Nrf2, Akt, p-Nrf2, and p-Akt were detected by Western blot with *β*-actin as the loading control. Values are the mean ± SE. The columns with the same superscript capital letters and with different superscript capital letters mean no significant difference (*P* > 0.05) and significant difference (*P* < 0.05), respectively.

To confirm the roles of Nrf2 in protecting IPEC-J2 cells against H_2_O_2_-induced oxidative stress, we silenced the Nrf2 gene expression by shRNA in IPEC-J2 cells, then treated Nrf2-knockdown cells with RSV and/or H_2_O_2_, and then determined cell viability, apoptosis, and expression of antioxidant genes. Knockdown of Nrf2 by shRNA largely eliminated the beneficial effects of RSV pretreatment based on the changes in cell viability and the expression of antioxidant genes. qRT-PCR analysis showed that knockdown of Nrf2 in IPEC-J2 cells sharply decreased cell viability (Figure 7(a)) and mRNA expression of SOD-1, CAT, claudin-1, occludin, and ZO-1 (Figures 7(b) and 7(c)) and abrogated protective effects of RSV on cellular apoptosis (Figure 7(d)) under oxidative stress. Western blot analysis showed that knockdown of Nrf2 significantly decreased the phosphorylation of Nrf2 (p-Nrf2/*β*-actin) compared with the control (SC group) under oxidative stress. However, the phosphorylation of Akt was not affected in Nrf2-knockdown cells compared with the control group (Figure 7(e)).

### 4.6. Role of PI3K/Akt on RSV-Induced Cytoprotection against Oxidative Stress

**Figure 8:**
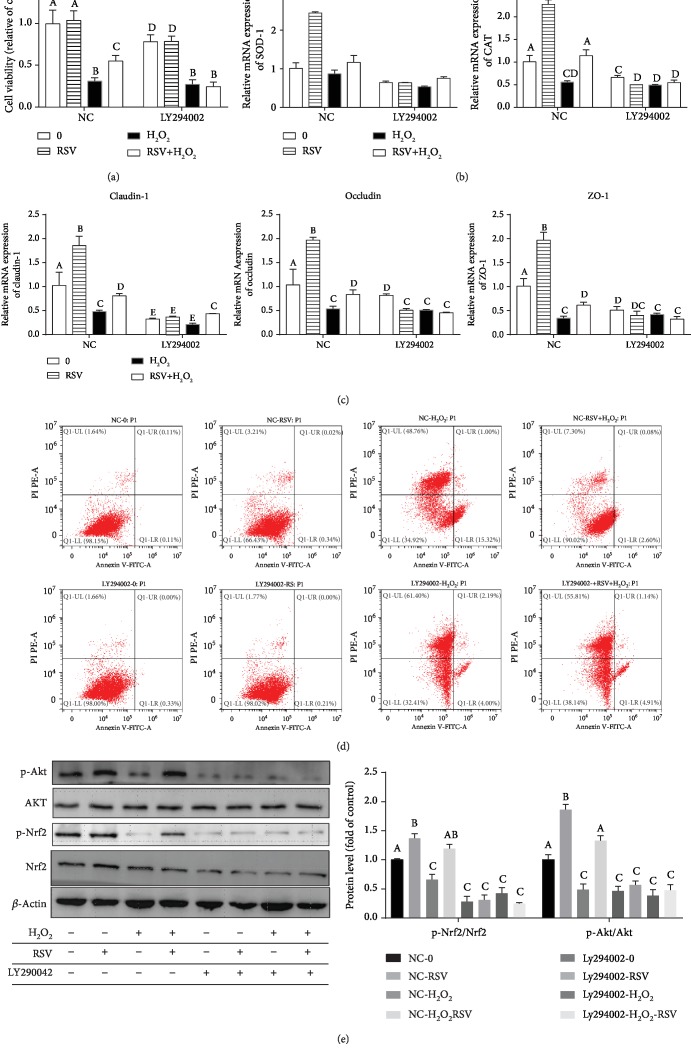
Effects of RSV and the selective inhibitor LY294002 on H_2_O_2_-induced cytotoxicity in IPEC-J2 cells. IPEC-J2 cells were pretreated with 25 *μ*M LY294002 for 2 h and then treated with 50 *μ*M RSV for 4 h before incubation with 500 *μ*M H_2_O_2_ for 4 hours. (a) Cell viability was measured using the CCK-8 assay. Results are presented as the percentage of cell viability compared with the control (0 *μ*M). (b, c) The relative expression of SOD-1, CAT, claudin-1, occludin, and ZO-1 was detected by qRT-PCR. Data are shown as ratios of abundance of target gene transcripts in the treated cells to those in the control cells after normalization to *β*-actin. (d) The apoptotic cells were analyzed by flow cytometry using Annexin V-PI double staining. (e) Protein levels of Nrf2, Akt, p-Nrf2, and p-Akt were detected by Western blot with *β*-actin as the loading control. Values are the mean ± SE. The columns with the same superscript capital letters and with different superscript capital letters mean no significant difference (*P* > 0.05) and significant difference (*P* < 0.05), respectively.

To examine the role of Akt in RSV-induced cytoprotection against H_2_O_2_-induced oxidative stress, we pretreated cells with LY294002, an inhibitor of PI3K/Akt, and then treated these cells with RSV and/or H_2_O_2_. We found that LY294002 abolished the protective effects of RSV against H_2_O_2_-induced downregulation of cell viability (Figure 8(a)), antioxidant genes (Figure 8(b)), and TJ protein levels (Figure 8(c)) and H_2_O_2_-induced apoptosis (Figure 8(d)). In addition, LY294002 treatment decreased the protein levels of Akt, Nrf2, p-Akt, and p-Nrf2 compared with the control (Figure 8(e)).

## 5. Discussion

The intestine is a major digestive and absorptive organ for nutrients and provides a selective barrier against endogenous and exogenous antigens. Numerous studies have revealed that intestinal health, especially the integrity of the intestinal barrier, plays a crucial role in maintaining an organism's healthy state. Oxidative stress is a critical factor involved in the intestinal barrier disruption in intestinal diseases. Therefore, identification of appropriate plant extracts as therapeutic agents to counteract oxidative stress-related diseases is a research hotspot at present. In this study, IPEC-J2 cells were exposed to H_2_O_2_ to induce oxidative stress. We found that H_2_O_2_ treatment resulted in the reduction of cell viability and TJ protein expression, overproduction of ROS, disturbance of oxidation and antioxidation, and, finally, induced apoptosis. Moreover, the results also showed that RSV significantly attenuated H_2_O_2_-induced cell injury through increasing cell viability and upregulation of the expression of antioxidant genes.

As a dietary supplement, RSV is a polyphenolic compound contained in various fruits and herbs. Ever since RSV was identified to have multiple biological functions (antioxidant, anti-inflammatory, anticancer, etc.), it has been paid great attention for practical applications [10, 11, 20–22]. In the present study, our results showed that resveratrol potently protected IPEC-J2 cells against oxidative stress-induced intestinal injury using lower toxicity. The small intestinal has been found to be the major site of RSV absorption and metabolism; thus, we hypothesized that the intestinal epithelium may be a potential target for the beneficial effects of RSV though the first-pass effect by the intestine and microbe. Meanwhile, the anti-inflammatory and antioxidative effects of RSV were studied in many cases, such as models of ischemia/reperfusion injury and LPS induction [23, 24]. However, according to modern medical perspectives, oxidative stress plays a crucial role in the progression of many diseases such as the intestinal inflammatory bowel. Intestinal integrity is the basic guarantee for intestinal function. Intestinal integrity relies on TJs, which are the principal determinants of intestinal barrier function that seal the paracellular space between neighboring epithelial cells [25, 26]. Occludin, claudins, and ZO-1 are major intestinal barrier proteins [27]. In the present study, the expression of ZO-1, occludin, and claudin-1 showed a significant reduction after H_2_O_2_ treatment, whereas RSV attenuated dysfunction of the intestinal barrier by upregulating the expression of TJ proteins under H_2_O_2_-induced oxidative stress. Moreover, the results showed that RSV significantly attenuated H_2_O_2_-induced cell damage through increasing cell viability and decreasing apoptotic rate in IPEC-J2 cells. It is easy to speculate that RSV attenuates intestinal injury and especially barrier disruption mainly by upregulating the oxidative status.

Oxidative stress is due to the imbalance between pro- and antioxidant factors. Organisms or cells have antioxidant systems, which involve SOD, catalase, and GSH peroxidase, to scavenge overproduced ROS. This study showed that RSV could upregulate the activities of catalase (CAT), SOD, and GSH peroxidase and maintain intracellular ROS homeostasis under H_2_O_2_-induced oxidative stress or normal condition. On the one hand, RSV can directly clear free radicals by upregulating antioxidant enzyme activity *in vitro* and *in vivo* [21, 28]. Wang and his colleagues showed that RSV can protect the Caco-2 cell against H_2_O_2_-induced oxidative damage by reducing the malondialdehyde level and intracellular ROS accumulation. RSV has been reported to improve mitochondrial biogenesis and redox status by upregulating SOD and GSH-Px activity in suckling piglets [29]. On the other hand, RSV can also upregulate the expression of antioxidant genes in many studies, which is consistent with our results. In the present study, RSV increased the expression of SODs, GSXs, and HO-1. In the intracellular antioxidant system, HO-1, SODs, CAT, and GPXs are phase 2 genes which are known as target genes mediated by the Nrf2/Keap1 signaling pathway. Meanwhile, many studies have revealed that RSV attenuates oxidative stress-induced intestinal dysfunction by upregulating HO-1 expression [30]. Upregulation of antioxidant genes is cellular adaptation to oxidative stress and is mainly or partly regulated by the Nrf2 pathway [31].

Nrf2, a pivotal sensor of oxidative stress in cells, is a transcription factor that plays a central role in the regulation of antioxidant and phase 2 detoxifying enzymes and related proteins [32]. Keap1 binds to Nrf2 in the cytoplasm and facilitates Nrf2 ubiquitination which can prevent Nrf2 translocation into the nucleus under physiological conditions [33]. When the levels of intracellular ROS sharply increase, overproduced ROS modifies the reactive cysteine residues on Keap1, and then Keap1 dissociates from Nrf2, leading to the translocation of Nrf2 into the nucleus and the expression of its target genes [33, 34]. Recent studies have revealed that the phosphorylation of Nrf2 facilitates Keap1/Nrf2 dissociation and Nrf2 nuclear translocation. In the present study, RSV upregulated the levels of p-Nrf2 and p-Akt in a dose-dependent manner, suggesting that RSV attenuates oxidative status likely by activating the Nrf2 signaling pathway. Many studies have revealed that RSV treatment dramatically upregulates the expression of Nrf2 and HO-1 *in vivo* and *in vitro* [35, 36]. Furthermore, RSV-induced upregulation of antioxidant genes and the intestinal epithelial cell barrier was inhibited by Nrf2 knockdown, suggesting that Nrf2 may be a major target of RSV in relieving oxidative stress. However, the phosphorylation levels of the Akt protein were increased upon RSV treatment in our study. The PI3K/Akt signaling pathway is commonly involved in the Nrf2-dependent transcription in diverse cell types of responding ROS insults [37, 38]. Han's study showed that chlorogenic acid protects MC3T3-E3 cells against oxidative stress through the PI3K/Akt-mediated Nrf2 signaling pathway to induce HO-1 expression, suggesting that Nrf2 may be a downstream signal target of PI3K/Akt [19]. Therefore, Western blot analysis showed that RSV-induced increase of the Nrf2 phosphorylation level was inhibited by the specific PI3K/Akt inhibitor LY290042. In addition, RSV-induced changes of cell viability, apoptosis, antioxidant genes, and TJ expression were also inhibited by the inhibitor. These results were similar to effects of Nrf2 knockdown on IPEC-J2 cells. Moreover, the phosphorylation level of Akt was not affected when Nrf2 was knocked down. It indicates that the PI3K/Akt pathway plays a pivotal role in the cytoprotective effects of RSV-induced Nrf2 activation against oxidative stress.

Collectively, the present findings provide important evidence of potential cytoprotective effects of RSV against H_2_O_2_-induced dysfunction in the intestinal barrier. Pretreatment with RSV protects IPEC-J2 cells against oxidative stress by promoting antioxidant systems' activities, decreasing intracellular ROS and the apoptotic rate, and upregulating the intestinal barrier. These cytoprotective effects of RSV at least partly depend on the PI3K/Akt-mediated Nrf2 signaling pathway.

## Figures and Tables

**Table 1:** Sequence of hairpin RNA.

Names	Sequence of hairpin RNA
shNRF2-sense	CCGGCATACTTTGGAGGCAAGATATCTCGAGATATCTTGCCTCCAAAGTATGTTTTTG
shNRF2-antisense	AATTCAAAAACATACTTTGGAGGCAAGATATCTCGAGATATCTTGCCTCCAAAGTATG

**Table 2:** Sequence of target gene primer.

Gene names	PubMed no.	Sequence of primer	Length (bp)
Claudin-1	XM_005670262.3	CTAGTGATGAGGCAGATGAA	250
		AGATAGGTCCGAAGCAGAT	
Occludin	NM_001163647.2	GAGTGATTCGGATTCTGTCT	181
		TAGCCATAACCATAGCCATAG	
ZO-1	XM_021098896.1	TTGATAGTGGCGTTGACA	126
		CCTCATCTTCATCATCTTCTAC	
Keap1	NM_001114671.1	TCTGCTTAGTCATGGTGACCT	158
		GGGGTTCCAGATGACAAGGG	
NFE2L2	MH101365.1	TGCAGCTTTTGGCAGAGACA	119
		AGGAGCAATGAAGACTGGGC	
SOD-1	NM_001190422.1	AAGGCCGTGTGTGTGCTGAA	279
		AGTGGCCACACCATCTTTGC	
HO-1	NM_001004027.1	TACCGCTCCCGAATGAACAC	209
		GTCACGGGAGTGGAGTCTTG	
CAT	NM_214301.2	CCTGCAACGTTCTGTAAGGC	72
		GCTTCATCTGGTCACTGGCT	
GSX-1	NM_214201.1	CCTAGCAGTGCCTAGAGTGC	143
		CGCCCATCTCAGGGGATTTT	
*β*-Actin	XM_003357928.4	TGCGGGACATCAAGGAGAAG	216
		AGTTGAAGGTGGTCTCGTGG	

## Data Availability

The data used to support the findings of this study are available from the corresponding authors upon request.
